# Automated concept and relationship extraction for the semi-automated ontology management (SEAM) system

**DOI:** 10.1186/s13326-015-0011-7

**Published:** 2015-04-02

**Authors:** Kristina Doing-Harris, Yarden Livnat, Stephane Meystre

**Affiliations:** University of Utah, Department of Biomedical Informatics, 421 Wakara Way, Suite 140, Salt Lake City, UT 84112 USA; Scientific Computing and Imaging Institute, University of Utah, Salt Lake City, UT USA

**Keywords:** Ontology, Natural language processing, Terminology extraction

## Abstract

**Background:**

We develop medical-specialty specific ontologies that contain the settled science and common term usage. We leverage current practices in information and relationship extraction to streamline the ontology development process. Our system combines different text types with information and relationship extraction techniques in a low overhead modifiable system. Our **SE**mi-**A**utomated ontology **M**aintenance (SEAM) system features a natural language processing pipeline for information extraction. Synonym and hierarchical groups are identified using corpus-based semantics and lexico-syntactic patterns. The semantic vectors we use are term frequency by inverse document frequency and context vectors.

Clinical documents contain the terms we want in an ontology. They also contain idiosyncratic usage and are unlikely to contain the linguistic constructs associated with synonym and hierarchy identification. By including both clinical and biomedical texts, SEAM can recommend terms from those appearing in both document types. The set of recommended terms is then used to filter the synonyms and hierarchical relationships extracted from the biomedical corpus.

We demonstrate the generality of the system across three use cases: ontologies for acute changes in mental status, Medically Unexplained Syndromes, and echocardiogram summary statements.

**Results:**

Across the three uses cases, we held the number of recommended terms relatively constant by changing SEAM’s parameters. Experts seem to find more than 300 recommended terms to be overwhelming. The approval rate of recommended terms increased as the number and specificity of clinical documents in the corpus increased. It was 60% when there were 199 clinical documents that were not specific to the ontology domain and 90% when there were 2879 documents very specific to the target domain.

We found that fewer than 100 recommended synonym groups were also preferred. Approval rates for synonym recommendations remained low varying from 43% to 25% as the number of journal articles increased from 19 to 47. Overall the number of recommended hierarchical relationships was very low although approval was good. It varied between 67% and 31%.

**Conclusion:**

SEAM produced a concise list of recommended clinical terms, synonyms and hierarchical relationships regardless of medical domain.

## Introduction

We created an ontology development system, **SE**mi-**A**utomated ontology **M**aintenance (SEAM) that leverages current practices in information and relationship extraction from text to streamline the process of generating knowledge structures. The knowledge structures that interest us are medical specialty-specific ontologies. We will use these ontologies for machine assisted clinical diagnostic decision support (CDS). CDS requires knowledge structures representing diagnostic criteria, a method for gathering patient information and the ability to reconcile the gathered patient information with the diagnostic knowledge structures. These requirements stem from diagnostic decision-making, which requires knowing at least two things: 1) the criteria for a diagnosis and 2) if this particular patient meets those criteria. The goal of the SEAM system is to facilitate the information acquisition necessary to construct ontologies that represent the *settled science* and *common term usage* with respect to either medical specialty or particular disease.

## Background

Our current approach to building diagnostic knowledge structures is to construct an application ontology of a specific disease or medical specialty. Here application ontology is used to differentiate them from domain ontologies like the one described in [1]. Ontology is an arrangement for defining concepts, the relationships between them, and rules relating to the combining of concepts and relations [2]. Concepts^a^ are roughly the “ideas” to which words refer (i.e. what the words “mean”), which is also called *semantics*. Concepts are often thought of as groups of semantically equivalent terms (e.g. *heart attack*, *myocardial infarction*, *MI*). These equivalences allow an automatic system to map terms used in one setting on to those used in another. Relationships between the terms are required because people often use terms that are semantically related, but not semantically equivalent, to represent the same idea. For instance, a clinician may in some situations refer interchangeably to *bowels* and *intestines*. For a machine to interpret these references, it must have a representation of the relationship between *bowels* and *intestines*. Rules are used to further refine when it is appropriate to invoke a relationship. Ontologies have been successfully used to identify semantic equivalence for database integration, text classification, translation, and other natural language tasks [3-8]. In our work ontology is a semantic stepping-stone between biomedical knowledge about disease and clinical knowledge about patients.

For us, three important tasks of application ontology development are: determining the settled science; finding common term usage; and establishing any idiosyncratic terms used in specific settings or by specific individuals. Determining the settled science is finding the concepts (ideas, entities, etc.) and relationships that have been agreed upon by the relevant scientific community. The settled science is reflected in the *common term usage* for that specialty. Term extraction focused on individual texts seems to conflate individual clinicians’ or authors’ use of common terms with settled science. The philosophical error here is mistaking epistemology with ontology, which is mistaking an individuals understanding of the world with the way the world is or at least an *agreed* upon understanding of the world. Individual textbooks and journal articles are not necessarily accurate with respect to the settled science or the breadth of commonly used terms, although the former is more likely than the latter. Clinical texts, in contrast, are likely to use a wider breadth of common terms, but reflect an epistemological understanding of the field. Their term use will derive from the common medical sublanguage for that specialty. Some individuals may also have idiosyncratic uses that are necessary to understand their writings, but would muddy a general ontology. Cognitive science puts these idiosyncratic terms in a separate vocabulary. Our approach to finding common usage that reflects settled science is to analyze both clinical records and biomedical information sources simultaneously in order to reconcile them during development.

### Semi-automatic ontology development and terminology management

Ontology induction has historically meant extracting ontological structure from text. On the one hand it seemed sensible that the use of language would reveal the concepts important to a domain and the linkages between them. On the other hand, that would only be sensible if the text’s author did not think the reader already had a shared, fundamental, base level understanding of the world. Buitelaar and Cimiano [9] call this most of the knowledge remaining “under the surface” of the text [9]. They point out that even with logical theories learned from text (e.g. Inductive Logic Programming), it is unclear how well those theories reflect the shared conceptualization that ontologies are designed to represent. The surface textual level does not correspond with the deeper semantic level. Due to this disconnect between text and semantics, concept and relationship identification from literature and chart review are currently, at best, semi-automated. Two types of expert consultation are necessary to verify the semantic content of the knowledge extracted from text. Domain experts are used to verify usage within the domain and ontology experts are used to verify the soundness of the knowledge structure.

Several systems for semi-automated ontology (or terminology) management have been developed [8,10-12] also in clinical medicine [13-18]. These systems all perform three basic functions: term, synonymy and relationship extraction on a targeted training corpus. Most of these ontology development systems are evaluated on the single domain for which they are created.

#### Term extraction

Term extraction techniques fall into two broad categories: Named Entity Recognition (NER) [8,10], and Information Extraction (IE) [12,16-19]. Aside from NER and IE, there are techniques that use corpus-based semantics, such as term frequency, inverse document frequency or other relative frequency information [8,20,21]. Other approaches focus on sentence-based semantics, such as context window, graph-based [15,22] or a combination of the two [23]. These semantic-based techniques require a predetermined set of terms to match. Text2Onto [13] for example uses both IE and corpus-based semantics. There are other systems, which require annotated text, that won’t be discussed here [14,24].

#### Synonym extraction

When looking for synonyms and short forms (i.e. acronyms and abbreviations) it is difficult to draw a clear line between term and relationship extraction [23]. Term matching using corpus-and sentence- based semantics can be thought of as synonym extraction as can the extraction of certain lexico-syntactic patterns (LSPs) (e.g. “also known as”).

#### Relationship extraction

Relationship information is extracted after the relevant terms have been found. The systems use the identified terms to locate relations between them. Relationships can be discovered by matching LSPs including those first described by Hearst [25] and those used by Banyex et al [16,17]. LSPs include “is a” for hierarchy and “(…)” or “also known as” for synonymy [25]. Other methods include hierarchical clustering [26-28] rules [8,10,23] and machine learning [12,22].

#### Corpora

As mentioned earlier people change the amount of explanation they include in a document depending on the expected audience. Therefore, it is sensible to use different corpora depending on the information you would like to find. One technique for ontology development is to extract terms from a target corpus and relationships from textbooks [10,16,17], journal articles or abstracts, [23,29], other sources such as Wikipedia [30], DBpedia [31], YAGO [32], WiBi [33] and data “forms” [34]. The terms and relationships can be combined during the extraction process [23] or in a manual post-process by human experts [17,35].

### Evaluation

Evaluation methodologies are many and varied. Term extraction is generally assessed with precision and recall, also called sensitivity and positive predictive value [22,35-38]. Precision and recall broadly refer to finding only the terms of interest and finding all of the terms of interest, respectively. Ontology development systems using this type of evaluation report high precision scores 70%-80% with lower recall around 50%. Others use the percentage of the recommended terms that are accepted by subject matter experts [8,39]. The approval scores for ontology development systems range from a low of 15% [35] to a high of 70% [38], with an average of around 60% [26,37].

Automatically comparing relationships is more complicated because it requires that matching terms are found [40]. If the terms are different, the system looking for matching relationships will not find them (e.g. car- > van vs. auto- > van). Also when comparing to an existing ontology or domain taxonomy [16,40] one must decide how to classify relationships that overlap, but do not match those in the existing ontology (e.g. bike- > tandem and bike- > unicycle vs. only bike- > tandem). For these reasons, relationship extraction is generally evaluated using approval rates judged on a scale of either three or five points from good to bad.

### Objective

The objective of this work is to develop a semi-automated ontology management system that can produce a concise list of recommended clinical terms, synonyms and hierarchical relationships. The innovation of the system is the combination of many term, synonym and relationship extraction techniques and the combined clinical and biomedical corpora to target the *settled science* and *common usage* for a particular medical domain (specialty or disease). In contrast to others, we evaluate the generality of the system across three use cases: Two hand-created ontologies (one for acute changes in mental status (ACMS) and one for Medically Unexplained Syndromes (MUS)); and an ontology of echocardiogram summary statements

### Implementation

**Figure 1:**
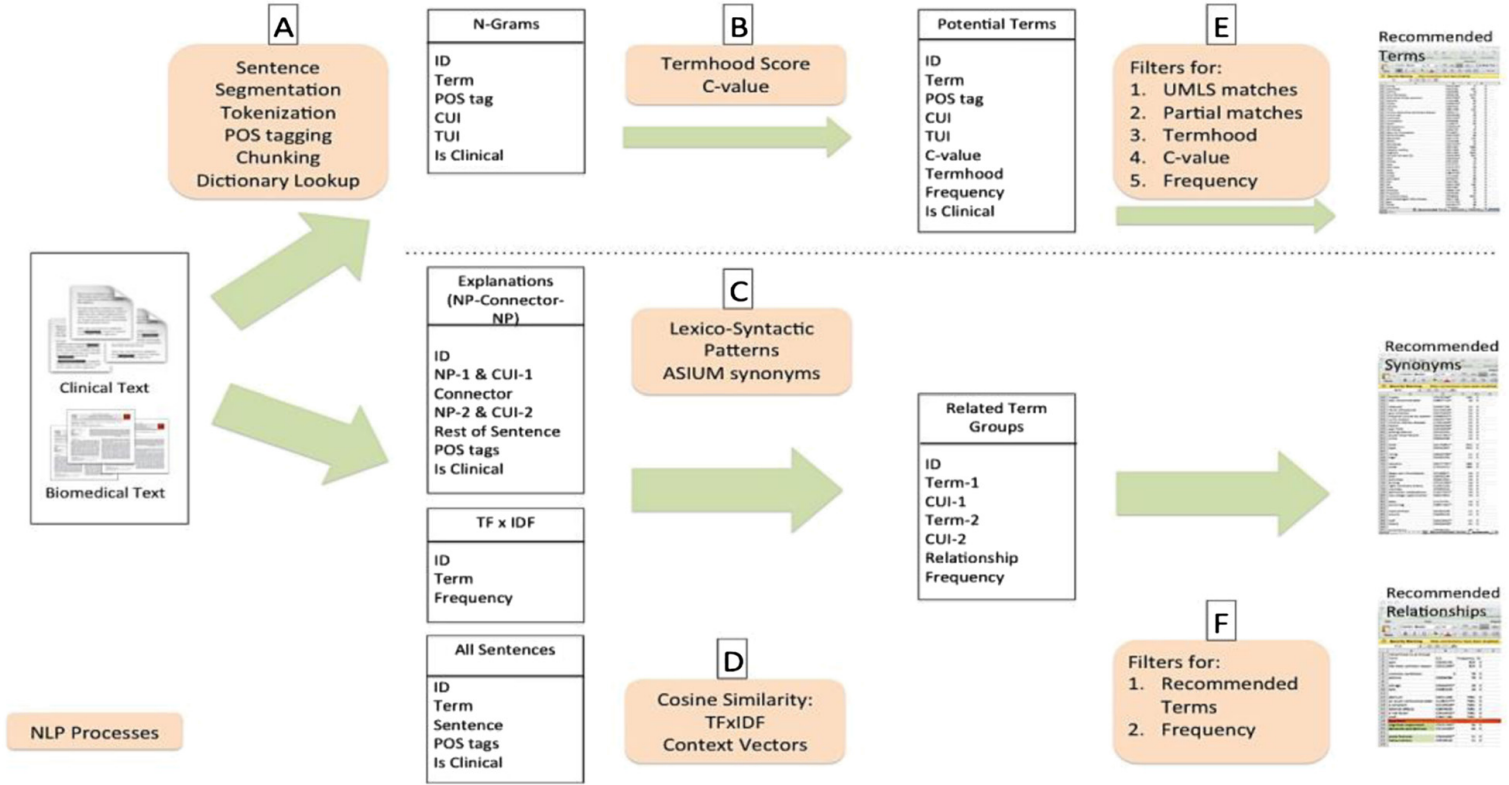
**A pictorial representation of the SEAM system.** This figure shows the three processing stages of the SEAM system. NLP processes are in the orange boxes. Each stage includes one or more phases **A…F**. Each box represents the database table created in that processing phase. The dotted line indicates that term processing is separate from relationship processing.

SEAM a stand-alone system that is configurable and modular. This self-sufficiency is an advantage because clinical records are held in protected environments that make the download and installation of external software packages difficult. SEAM is built as a Java project with modular processing steps and separate processing streams for terms and relationships. We incorporate many of the different approaches to term and relationship finding. The idea is to collect as much information as possible and then to use the combined corpus and filtering parameters to increase precision without sacrificing recall. <H2 > The SEAM structure is depicted in Figure 1.

#### Pre-preprocessing

Pre-processing (Figure 1, phase A) identifies the component parts of a text so that those parts may be used in text processing. The component parts identified begin with tokens. The process is called tokenization. Tokens can be single words or punctuation symbols. Tokens are used in *sentence segmentation* to identify sentences boundaries. POS tagging attaches a part-of-speech tag to each token. Chunking gathers tagged tokens into noun phrases and verb phrases. SEAM uses the OpenNLP toolkit, as described in detail in [41]. We chose not to stem the tokens because the UMLS Metathesaurus contains lexical variants for each concept. We estimated that stemming would not provide an increase in performance.

The output of this pre-processing is the structures that we will use in the remaining processing phases. First we find multi-word terms that the dictionary lookup may have missed. The smallest term we are interested in is a single word term, also called a unigram. As the number of words in a term increases it is referred to as a 2-gram, 3-gram, etc. An example of a unigram is “aorta” and a 4-gram is “obstructive coronary artery disease”. Terms with an unknown number of words are called N-grams. We are interested in N-grams up to the size of a noun or verb phrase.

We add dictionary lookup to pre-processing because we are interested in medical terms. The dictionary look-up uses an Apache Lucene index built by selecting all terms from the clinical terminology sources and with the disorder semantic types, listed in Table 1, from the Unified Medical Language System (UMLS) Metathesaurus [42]. Since we do not pre-process words from the document (e.g. stemming), we do not pre-process the UMLS Metathesaurus terms. We use lookup to find the UMLS Metathesaurus concept unique identifiers (CUI) and semantic type identifiers (TUI) for fully and partially matched N-grams. Fully matched N-grams appear exactly in the UMLS Metathesaurus. If the Lucene index finds more that one match for a given string, the highest scoring matched concept is selected. Ties are not arbitrated rather the first match returned by the index is used. Partially matched N-grams contain at least one word in common with a UMLS concept. Partial matches are further restricted to the desired semantic types listed in Table 1. Partial matches are selected based on the match with the highest number of words in common and the fewest extra words. Our Lucene query does not respect word order. Again, the first match returned by the Lucene index, with the stated criteria, is chosen.

**Table 1:** **The UMLS semantic types used by SEAM for partial matches and final recommendations**

T020	Acquired Abnormality
T190	Anatomical Abnormality
T049	Cell or Molecular Dysfunction
T019	Congenital Abnormality
T047	Disease or Syndrome
T050	Experimental Model of Disease
T037	Injury or Poisoning
T048	Mental or Behavioral Dysfunction
T191	Neoplastic Process
T046	Pathologic Function
T184	Sign or Symptom
T033	Finding
T029	Body Location or Region
T080	Qualitative Concept
T023	Body Part, Organ or Organ Component
T081	Quantitative Concept

The second text component we are interested is called an *explanation. Explanations* are patterns of phrases within a sentence. The pattern of an *explanation* is noun phrase, *connector*, and noun phrase. A *connector* is any combination of tokens, such as a verb phrase or a parenthesis. Examples of explanations are “CAM or CAM ICU is a highly sensitive and specific method…” and “a-fib on Coumadin…” The noun phrases in these examples are “CAM or CAM ICU,” “a highly sensitive and specific method,” “a-fib,” and “Coumadin.” The *connectors* are “is” and “on.” A single sentence may contain more than one *explanation*.

The final component of interest is a term frequency by inverse document frequency (TF-IDF) vector for each N-gram. The TF-IDF vector contains one entry for each document. An entry is the frequency of occurrence of the N-gram in that document normalized by the inverse of its average frequency across all documents. These vectors are compared for similarity.

#### Term extraction

In the term extraction phase (Figure 1, phase B), we identify the terms that are relevant to the use case at hand by locating health-related terms in the corpus. Dictionary lookup will have already found many health-related terms. The c-value and Termhood scoring algorithms find health-related terms that may have been missed. The c-value and Termhood equations are listed in Table 2 and are described in more detail in [39].

**Table 2:** **Equations used in SEAM**

C-value (a) [18]	$$ \left\{\begin{array}{l}\kern12.5em lo{g}_2\left|a\right|\cdot f(a),\kern2em \left|\alpha\ is\ not\ nested\right.\hfill \\ {}lo{g}_2\left|a\right|\left(f(a)-\frac{1}{P\left({T}_{\alpha}\right)}{\displaystyle \sum_{b\epsilon {T}_{\alpha }}f(b)}\right),\kern1em \left| otherwise\right.\hfill \end{array}\right. $$
	where:
	$\alpha$ is the candidate string
	*f(.)* is its frequency of occurrence in the corpus
	Τ_a_ is the set of extracted candidate terms that contain a
	*P*(Τ_a_) Is the number of these candidate terms
Termhood (a) $\log \left(\frac{P\left( vote= yes\right)}{P\left( vote= no\right)}\right)$ [53]	= −0.7836 +
	0.7541* FirstPOS _ ADJECTIVE –
	1.3722* FirstPOS _ ADVERB +
	0.3541* FirstPOS _ NOUN +
	1.4182 * FirstPOS _ VERB –
	0.7722 * LastPOS _ ADJECTIVE +
	2.2576 * LastPOS _ ADVERB +
	0.0285 * LastPOS_NOUN +
	0.6038 * LastPOS _ VERB +
	1.2899 * NP _ VALUE +
	1.0475 * REPEAT _ SUP _ GREATER _ MEDIAN +
	0.8417 * REPEAT _ SUB _ GREATER _ MEDIAN +
	0.8422 * DISTINCT _ PERHOST _ GREATER _ THAN _ MEDIAN
	where:
	POS is Part of Speech tag
	REPEAT_SUP is number of supra (candidate terms containing a) = *P* (Τ_a_)
	REPEAT_SUB is subgroup (candidate terms that are contained within a) = P (Α_t_)
	NP_VALUE is a a noun phrase
	DISTINCT_PER_HOST is equivalent to document frequency
	MEDIAN is calculated for the whole document set
TF-IDF = w_i,j_ = TF_i,j_ x IDF_i_ [43]	$T{F}_{i,j}=\frac{f_{i,j}}{ma{x}_z{f}_{z,j}}$
	where:
	TF_i,j_ is term frequency for keyword k_i_ in document d_j_
	f_i,j_ is the number of times k_i_ appears in d_j_
	max_z_f_z,j_ is the maximum frequency across all keywords k_z_ in d_j_
	$$ ID{F}_i= log\frac{N}{n_i} $$
	where:
	IDF_i_ is the inverse document frequency for keyword k_i_
	N is the total number of documents in the corpus
	n_j_ is the number of documents that k_i_ appears in
Cosine similarity [43] $cosine\left(\overrightarrow{w_c},\overrightarrow{w_s}\right)=\frac{\overrightarrow{w_c}\cdot \overrightarrow{w_s}}{\overrightarrow{w_c}\times \overrightarrow{w_s}}$	$=\frac{{\displaystyle {\sum}_{i=1}^K}{w}_{i,c}{w}_{i,s}}{\sqrt{{\displaystyle {\sum}_{i=1}^K}{w}_{i,c}^2}\sqrt{{\displaystyle {\sum}_{i=1}^K}{w}_{i,s}^2}}$
	where
	w_i,j_ is defined above

Stop words are eliminated in this phase, since they were not removed during the text search phase. Stop words within noun and verb phrases were retained to aid in relationship identification. Specifically, determiners were not removed because nouns joined by “is a” are important for finding hierarchical relationships.

#### Synonym extraction

In this phase, synonym groups are extracted (Figure 1, phase C). These groups reflect the concepts relevant to the use case. We use four synonym extraction techniques that fall into two related pairs. The first pair of techniques determines potential groupings of topics that correlate across the whole corpora. The second pair uses pattern matching on the *explanations*.

The corpus-based semantics filters are TF-IDF vectors [43] and context vectors [44-47] with above threshold similarity. We define a context vector as the sequence of ten words that occurred in the text, 5 before and 5 after the target term. Each term has an array of words for each of the 10 positions containing the words that occurred in that position. The dot product of two such vectors is the number of times they have at least 1 word in the same position. Preliminary tests found that position agnostic comparisons produced too many false positives.

The pattern matching pair of synonym extraction techniques operate on the *explanations*. First we use lexico-syntactic patterns (LSPs) [25]. LSPs are patterns of words and symbols that appear as the *connector* in an *explanation* (e.g., NP1 *also known as* NP2, NP1 (NP2, and NP2 *referred to as* NP1). The LSPs are listed in Table 3. A second pattern overlaps with the first but includes a verb and a match to NP2 as well. NP1s with the same connector that contains a verb and the same NP2 are likely to be synonymous [22,46,47]. For example, the *explanations* “a pressure pain (NP1) associated with (connector) diaphoresis (NP2)” and “substernal chest pressure (NP1) associated with (connector) diaphoresis (NP2)” indicates that *a pressure pain* and *substernal chest pressure* are synonymous.

**Table 3:** **Lexico-syntactic patterns used to identify relationships** [25]

**Relationship**	**Patterns found between NP1 and NP2**
Synonymy	“%also%known%as%”, “(“, “aka”, “so called”, “also called”, “%also% referred% to%”, “%referred% to%”
Hierarchy	“%such%as%”, “%or other%”, “%and other%”,”%including%”, “%associated with”, “is”, “are”, “is “, “%type of%”,”are “

#### Relationship extraction

This phase (Figure 1, phase D) identifies additional hierarchical relationships using LSPs (see Table 2). These LSPs are designed to identify hierarchical relationships (e.g. *is a*, *a type of*, *such as*) [25].

#### Candidate filtering

Recommendation lists cannot be too long or reviewers will find them overwhelming so we filter them to find a restricted set with high precision (Figure 1, phase E and F). In this phase the final lists of recommended terms, synonyms and hierarchical relationships are produced.

We exclude terms that are so uncommon that they are unlikely to be relevant or so likely that they are probably not specific to the topic at hand, by focusing on term that occurs in between 5% of the number of documents in the smaller of the two corpora (with a minimum value of 1) and 95% of the number documents in the larger of the two corpora. Inverse document frequency is designed to do this, but since we are combining corpora we found this augmentation necessary.

We also focus on terms that occurred above a frequency threshold across both corpora. This threshold is discussed in the results of each use case because it changes for each one.

We employ four term filters that fall into two pairs. Filters one and two identify direct and partial UMLS Metathesaurus matches with the semantic types from Table 1. Filters three and four find non-matched terms with c-value and Termhood scores also above threshold. Terms that share the same UMLS CUI are combined into a synonym group for presentation together.

Filters for term relationship groups, focus on groups that contain recommended terms from phase E, with frequency counts and the method used to find the group. Here frequency is the number of times the relationship was found. The settings for each of these filters are described in the evaluation sections as they change depending on the use case.

#### System parameters

The configurable parameters for SEAM are semantic cosine similarity thresholds (TF-IDF and context vector) and term filter settings. Parameters are configured to produce the number of results found in preliminary testing to be comfortable for experts to review (around 200) and a valid term percentage that looked promising. We will include the parameter configuration with the description of each implementation.

We observed that cosine similarity thresholds of 0.90 and 0.80 for TF-IDF and context, respectively, performed consistently well. These parameters did not need to change with the size of the corpora because TF-IDF is scaled in the number of documents and our implementation of context vectors did not take into account the number of times context words overlapped.

SEAM’s configurable parameters are 1) frequency threshold for UMLS Metathesaurus full matches; 2) frequency threshold for UMLS Metathesaurus partial matches restricted to the relevant semantic types; 3) frequency threshold for terms not found in the UMLS Metathesaurus; 4) C-value threshold; 5) Termhood score threshold. The choice of C-value and Termhood thresholds is based on the point at which the number of terms found drops below 50. The precision of these methods is low so allowing larger numbers of terms through the filters reduces precision. In preliminary testing, we determined that with returns above 50 the depletion was unacceptable.

#### Evaluation criterion

The goal of our system is to recommend terms in common usage. To that end, we consider a term recommendation successful if two out of our five reviewers find it relevant to the use case.

### Use cases

We apply the SEAM system to three use cases. The first two use cases are ontology expansions. Terms identified by SEAM are compared with concepts currently included in these two disorder-specific ontologies. The comparison is used to refine the input and configuration of SEAM to produce concise lists for expert approval, not as “gold standards” against which SEAM is being judged.

#### Use case 1

We will use SEAM to identify new terms and relationships for an ontology of acute changes in mental status (ACMS). The existing ACMS ontology was built using terms extracted from hand-reviewed clinical documents from 25 patients, 12 of whom were judged to have delirium. Two subject matter experts located 168 terms. Those terms were used to construct the ontology in OWL. Terms may be associated with more than one concept. For example, delirium can be both a syndrome and a symptom. They can also indicate higher-level concepts to include. The resultant ontology has 195 concepts (160 classes and 35 individuals) with 369 hierarchical relationships.

#### Use case 2

We will be using the same process as Use Case 1 to find new terms and relationships for an existing ontology of medically unexplained syndromes (MUS). The existing ontology was created using: reference to the ICD 11 - body systems ontology [48]; consultation with domain experts on inclusion and exclusion criteria for the three most common MUS (irritable bowel syndrome, chronic fatigue syndrome and fibromyalgia), literature and chart reviews. It was constructed in accordance with the ideas on syndrome domain ontology described in Doing-Harris [1]. It is build in OWL. The ontology contains 236 entities (201 classes and 36 individuals) and 413 hierarchical relationships.

#### Use case 3

The third application of SEAM will be to enhance the pick-list of findings used to summarize echocardiogram (ECHO) reports. The pick-list is a hierarchical smart list of relevant sentence pieces that the hospital EHR system has to facilitate the creation of echocardiogram summary reports. Clinician’s pick pieces from the list to create summary sentences. Hence it is called a pick-list. Each summary report includes a list of clinically relevant findings. Choosing entries from a predetermined pick-list populates this summary list. The cardiologists at the University of Utah would like to increase the number of entries on the pick-list. To evaluate SEAM, we will determine the number of current pick-list sentences in which SEAM identifies the relevant terms as well as the number of novel relevant terms and relationships identified by SEAM.

### Corpora

#### Clinical corpora

The corpora used to evaluate SEAM differed within and between the three use cases. These differences allowed us to investigate the impact of corpus changes on the lists of recommended terms, synonyms and hierarchical relationships returned. For the ACMS and MUS use cases, we employed clinical documents from the 2009 i2b2 Medications Challenge [49]. The first set was a selection of 199 of the i2b2 text only documents. The second increased the selection to 696 documents. For the ECHO application, the clinical documents were 2879 echocardiogram reports generated by the University of Utah department of cardiology. Since we were only interested in summary statements, we excluded all other portions of the reports. We also combined each set of five summary statements into a single document to reduce the number of columns in the TF-IDF database table. This amalgamation will affect the IDF values for the terms, but will do so equally for all terms so it should not affect results.

#### Biomedical corpora

The specificity of a corpus to a domain rested on the selection of journal articles for use cases 1 and 2. Journal articles (in PDF format) were used for the ACMS and MUS use cases. These articles were collected during literature reviews for papers and grant proposals related to the two topics. For the ACMS use case we included 19 articles. For the MUS use case we included 47 articles. For the ACMS and ECHO use cases we also selected from a database of case reports downloaded automatically from PubMed (as XML files). These reports were chosen by the medical specialties listed in PubMed. They were neurology and cardiology, respectively. Their body text was isolated for analysis.

## Results

Results are reported in terms of direct and partial matches to the original ontologies. These match types are calculated the same way as the direct and partial matches discussed earlier with the UMLS Metathesaurus. Direct matches are as they sound. Partial matching means that the recommended terms share a word or words with the matched entry. For example, the recommended term *hallucinations* is found in the ontology term *visual hallucinations* or (less helpfully) *commands* is found in *able to follow commands*. In the other direction, recommended terms incorporate terms from the ontology. For example the recommended term *permanent cognitive decline and dementia* partially matches the ontology concept *dementia*. A more detailed look at the difference between direct and partial matches including measures of term overlap would be interesting in terms of each individual ontology, but we did not think that the its reflection on system performance is enough to bring it within the scope of this paper.

Direct or partial term matches between the recommended terms and the existing ontologies were found using a query for term equivalence and overlap. Experts did not review this determination. Five experts reviewed results for each use case for a total of 7 medical doctors, a third year medical student, and the first author. They performed the expert reviews of the recommended terms that were not found in the existing ontologies.

Term extraction included terms with the UMLS semantic types *Qualitative Concept* and *Quantitative Concept*. The UMLS definitions are “A concept, which is an assessment of some quality, rather than a direct measurement“ and “A concept, which is an assessment of some quality, rather than a direct measurement” respectively [50]. These terms are necessary for ontology development, but are not specific to a domain. They include terms like *borderline*, *identified*, *revealed*, and *improved*. These terms were excluded from expert review because they are not domain specific.

### Use case 1: ACMS ontology terms and relations extraction

*A large targeted clinical corpus (199 files from i2b2), with added biomedical articles (19)*: a corpus of 199 clinical records that contained the words *neuro*- or *mental status* were selected from the i2b2 dataset of 696 records.

This configuration generated 39,863 potential terms. The breakdown of terms found within the corpus is listed in Table 4.

**Table 4:** **The breakdown in potential terms found from each corpus with those found in the existing ontology and matched to the UMLS Metathesaurus**

**Use case**	**Potential terms**	**Terms from each Corpus**			**Existing ontology terms found**		**Matched to UMLS**	
							**Metathesaurus**	
		**Biomedical**	**Clinical**	**Overlap**	**Full**	**Full+ Partial/Total**	**Full**	**Partial**
**ACMS**	39,863	14,407	23,936	1,520	66	163/166	1,138	8,021
**MUS**	86,931	27,152	56,883	2,896	88	219/219	4,962	18,410
**ECHO**	83,368	78,358	4,268	1,342	198	198/198	4,695	15,843

Term filter settings: All four term filters’ frequency thresholds were set to 10 occurrences. Filters one and two were for UMLS full and partial matches with the relevant semantic types. Filters three and four found non-matches with C-value above 50 and Termhood above 4.6, none of these terms were relevant so they are ignored in the expert analysis.

After filtering there were 173 recommended terms. The number of recommendations from each filter is listed in Table 5. We consider the 25 recommended terms found in the ontology to be successful recommendations because we are interested in how the system performed not in the ontology coverage. We use the existence of a term in the ontology only as an indication that it is relevant to the topic, and therefore an accepted recommendation.

**Table 5:** **SEAM term results for the ACMS targeted clinical corpus (n = 199), with 19 biomedical articles**

	**Filter**	
**Terms**	Filter 1: Direct UMLS matches in both corpora	81
	Filter 2: Partial matches to UMLS in both corpora	101
	Filter 3: Non-matches with c-value > 50 in clinical corpus or both	-
	Filter 4: Non-matches with Termhood score > 4.6 in clinical corpus or both	-
	Combined recommended terms with the same CUI	-9
	**Recommended Terms (total)**	**173**
**Found in ontology**	Fully Matched	12
	Partially Matched	13
	**Recommended Terms found in ACMS Ontology**	**25**

Synonym and Hierarchy Filter Settings: The frequency threshold for synonyms was set to ten. We observed that synonym and hierarchy groups identified using LSPs were few in number, but contained more accepted relationships. ASIUM synonym groups were the same. Therefore, we lowered the threshold to one occurrence for relationships identified by these techniques, leaving it at 10 for TF-IDF and context identified synonyms.

These settings found 51 synonym groups. All groups contained recommended terms and terms that had matched to UMLS (full or partial). The breakdown of relationship groups from the different filters is in Table 6. Hierarchical Relationship Filters returned six relationship groups.

**Table 6:** **SEAM relationship results for the ACMS targeted clinical corpus (n = 199), with 19 biomedical articles**

	**Filter**	
**Relationships**	Filter 1: TF-IDF	16
	Filter 2: LSP	21
	Filter 3: ASIUM	14
	Filter 4: Context Vectors	-
	**Recommended Synonymy Groups (total)**	**51**
	**Recommended Hierarchy Relationships (total)**	**6**

#### Expert review

Results of the expert reviews of recommended terms across use cases are reported in Table 7. For the non-matched recommended terms, agreement between reviewers reflected the differences in language use discussed earlier. The Fliess’ kappa score across the five experts was 0.38 showing only fair agreement. The number of terms considered related to ACMS varied between 19 and 46. Forty-eight terms were found by two or more reviewers to be relevant to ACMS but not in the existing ontology and 31 were qualitative terms, which are relevant but had not been considered when the ontology was constructed, this is an approval rate of 60%. Leaving 70 recommended terms as unrelated to ACMS, 23 accepted by only 1 reviewer and 47 rejected by all 5. The maximum approval rate would be 73%, but since our goal is generality, not idiosyncrasy, we consider 60% the true approval rate (see Table 7).

**Table 7:** **Expert review of terms for each use case, Recommended vs. Accepted terms. Qualitative terms are reported separately because they were not considered when the ontologies were first constructed**

**Use Case**	**Reco-mmended**	**Matched**	**Qualitative**	**Accepted (2 + revs)**	**Total Accepted**	**Accepted** **(<2 revs)**	**Misses**	**Fleiss’ Kappa**
**ACMS**	173	25 (14%)	31 (18%)	48 (28%)	103 (60%)	23 (13%)	47 (26%)	0.38 (Fair)
**MUS**	271	61 (23%)	35 (13%)	67 (25%)	163 (60%)	26 (9%)	83 (31%)	0.29 (Fair)
**ECHO**	363	289 (80%)	N/A	37 (10%)	326 (90%)	14 (4%)	23 (6%)	0.66 (Substantial)

Results of the expert reviews of recommended relationships (across use cases) are reported in Table 8. For synonyms, we found eighteen approved synonym groups, 43%. For the hierarchical relationship groups, all five experts agreed that four of the six groups, 67%, contained valid relationships relevant to ACMS, two relationships from the current ontology and two new relationships.

**Table 8:** **Expert review of relationships for each use case, Recommended vs. Accepted relationships**

**Use Case**	**Type**	**Rec**	**Approved**	**Misses**
**ACMS**	Synonymy	51	**18 (43%)**	33 (57%)
	Hierarchy	6	**4 (67%)**	2 (33%)
**MUS**	Synonymy	75	**19 (25%)**	56 (75%)
	Hierarchy	14	**9 (64%)**	5 (36%)
**ECHO**	Synonymy	127	**34 (27%)**	93 (73%)
	Hierarchy	16	**5 (31%)**	11 (69%)

#### Alternative corpus constructions

For this use case we started with a small general clinical corpus (76 files from i2b2) and added biomedical articles (19). However, even with changes in the filters we found the ontology coverage low. This clinical document corpus was small and heavily weighted toward cardiac cases. Thirty-four out of 76 files contained the string *cardi*-, while only 19 contained *neuro-* or *mental status*. We hypothesized that this composition was impeding the identification of the ontology terms. Therefore, we altered it to the configuration above.

In a second alternative corpus construction we retrieved case reports from the PubMed case reports download database described earlier, rather than use biomedical articles. One of our colleagues reasoned that case reports may contain language that more closely resembled the language used in clinical documents. The possible language resemblance and ability to directly target reports containing three ACMS key terms (delirium, delirious and altered mental status) promised to return a more concise list of terms. Unfortunately, this did not happen. The number of recommended terms increased to 255, but the number of ontology terms directly and partially matched decreased to 33.

### Use case 2: MUS ontology comparisons

#### Large clinical corpus (696), with 47 biomedical articles

The MUS ontology contains 219 concepts. It is designed differently to the ACMS ontology. Because MUS is a diagnosis of exclusion, the ontology includes many other diagnoses to exclude. The contributions of each document type, the matches to the existing ontology, and UMLS Metathesaurus are listed in Table 4.

The filters remained the same as in the previous use case except the occurrence threshold was increased to 20 and non-matched terms were restricted to only those appearing in both corpora. After filtering there was a total of 271 recommended terms. Table 9 shows the number of recommended terms that passed each filter and the matches to the current ontology. Sixty-two recommended terms are either full or partial matches the current ontology.

**Table 9:** **SEAM term results for the MUS large clinical corpus (n = 696), with 47 biomedical articles**

	**Filter**	
**Terms**	Filter 1: Direct UMLS matches in both corpora	134
	Filter 2: Partial matches to UMLS in both corpora	148
	Filter 3: Non-matches with c-value > 50 in both corpora	5
	Filter 4: Non-matches with Termhood score > 4.6 in both corpora	2
	Combined recommended terms with the same CUI	-18
	**Recommended Terms (total)**	**271**
**Found in ontology**	Fully Matched	12
	Partially Matched	50
	**Recommended Terms found in MUS Ontology**	**62**

Table 10 shows the breakdown of filter results for the 75 synonym groups that were recommended. TF-IDF performed badly so it was not used to generate synonym groups. Fourteen hierarchical relationships were recommended.

**Table 10:** **SEAM relationship results for the MUS large clinical corpus (n = 696), with 47 biomedical articles**

	**Filter**	
**Relationships**	Filter 1: TF-IDF	-
	Filter 2: LSP	40
	Filter 3: ASIUM	19
	Filter 4: Context Vectors	16
	**Recommended Synonymy Groups (total)**	**75**
	**Recommended Hierarchy Relationships (total)**	**14**

#### Expert review

Results of the expert reviews of recommended terms across use cases are reported in Table 7. Expert agreement, by Fliess’ kappa, is lower than for ACMS although still fair at 0.29. Experts found between 8 to 58 terms relevant. We used agreement between 2 reviewers as an assessment of relevance. As you can see in Table 7, twenty-five percent of the recommended terms were approved by more than one reviewer as related to MUS, but are missing from the ontology. Thirty-five terms are from the UMLS semantic types *Qualitative* and *Quantitative Concepts* and were not explicitly considered in building the MUS ontology. Adding together the matched, qualitative, and related terms, we estimate the approval rate at 60%.

We had an additional point of comparison for this dataset, a list of MUS symptoms identified by another group within the project. Since the synonym list was not designed as an ontology we used it as an indicator of whether a term was useful in identifying MUS, not as another ontology for coverage assessment. Thirty-three (33) of the 62 recommended terms that matched to the existing ontology also matched to the symptom list. Nineteen other recommended terms also matched to the symptoms list. Nine of the terms considered misses by expert review were on the symptom list. These 9 are not considered approved terms because they likely represent idiosyncratic use. The symptoms list is not comprehensive, as it does not include 28 of the 61 terms matched to the current ontology; including *chronic pain*, *fibromyalgia*, and *constipation*. Two of the qualitative terms are included in the symptoms list, indicating the utility of at least some of these terms.

Results of the expert reviews of recommended relationships across use cases are reported in Table 8. After excluding the TF-IDF groups, SEAM recommended 75 synonym groups. LSPs created the largest number of synonym groups, probably due to the large number of documents. Expert review of the synonyms and relationships found nineteen synonym groups (25%) contained valid synonyms. Nine of the 14 hierarchical groups (64%) were considered correct by 1 or more of the expert reviewers.

These results are similar to the best configuration for the ACMS ontology, except that there were more synonym groups and their approval rate was lower. Table 10 shows the comparison of SEAM configurations.

### Use case 3: echocardiogram summary statements

#### Targeted extremely large clinical corpus (2874) with a large number of case reports (232)

In this use case we first wanted to identify the pick-list sentences. We found 1174 unique sentences in the clinical (summary) documents. By looking for sentences that share their first 200 characters and occurred in more than 5 documents, we identified that 131 sentences were confirmed to have come from the pick-list. Our goal is to produce a recommended term list that contains at least one term from each of the 131 pick-list sentences as well as terms related to echocardiography not in the pick-list with a few distractors as possible. There were 198 of these pick-list terms. The breakdown of terms from the clinical, biomedical, the pick-list terms (called existing ontology, here) and UMLS Metathesaurus are listed in Table 4. Before filtering, SEAM finds 198 distinct pick-list terms that are associated with 131 pick-list sentences 93%.

Term filter settings are summarized in Table 11. For this use case the frequency threshold was reduced to five, due to the relative small number of terms from the clinical corpus. All four term filters were also set to include terms that appear only in the clinical corpus as well as those from both. The repetitions nature of these terms is indicated by the high number of repeat CUIs found (70).

**Table 11:** **SEAM term results for the Echocardiogram large clinical corpus (n = 2874/5 = 575), with 232 case reports**

	**Filter**	
**Terms**	Filter 1: Direct UMLS matches in both corpora	90
	Filter 2: Partial matches to UMLS in both corpora	180
	Filter 3: Non-matches with c-value > 50 in both corpora	71
	Filter 4: Non-matches with Termhood score > 4.6 in both corpora	89
	Combined recommended terms with the same CUI	-70
	**Recommended Terms (total)**	**360**
	**Recommended Terms found on the Pick List**	**289**

Table 12 shows the breakdown of synonym groups found by the different methods. The majority are from TF-IDF, which is currently our least accurate algorithm.

**Table 12:** **SEAM relationship results for the Echocardiogram large clinical corpus (n = 2874/5 = 575), with 232 case reports**

	**Filter**	
**Relationships**	Filter 1: TF-IDF	66
	Filter 2: LSP	29
	Filter 3: ASIUM	25
	Filter 4: Context Vectors	1
	**Recommended Synonymy Groups (total)**	**121**
	**Recommended Hierarchy Relationships (total)**	**16**

#### Expert review

Results of the expert reviews of recommended terms across use cases are reported in Table 7. Eighty percent of the recommended terms match 152 of the distinct pick-list terms. Because we include partial matches, more than one recommended term could match the same pick-list term. For this task we did not exclude qualitative terms. Expert agreement on this recommended term list was substantial (Fliess’ kappa = 0.66), with relevant term lists ranging in size from 42 to 26. Two or more reviewers agreed on 10% of the terms. Combining pick-list and agreed upon terms results in an approval rate of 90% (see Table 7).

Results of the expert reviews of recommended relationships across use cases are reported in Table 8. The synonym and relationship filters are the same as used in the previous two use cases. They return 127 synonyms and 16 hierarchical relationships. Repeated CUIs identified 70 synonymous relationships. Only 4 of the 5 expert reviewers assessed the hierarchical relationships. Twenty-seven percent of the synonymy relations were considered valid by at least 1 reviewer. Thirty-one percent of the relationships were found to be valid by more than two reviewers. Forty-four percent of the 16 hierarchical relationships were found to be valid by at least one reviewer, while twenty-five percent had three or more.

## Discussion

We found that SEAM produced a concise list of recommended ontology term additions across a variety of use cases. The approval rate of recommended terms increased with the number and specificity of clinical documents in the corpus, from 53% when there were 199 clinical documents that were not specific to the ontology domain, to 89% when there were 2879 documents very specific to the target domain.

Approval rates for relationship recommendations remained relatively stable as the number of journal articles increased from 19 to 47 at around 60%. Changing to case reports caused the approval rate to drop to around 30% despite a substantial increase in the total number of documents. Overall the number of recommended hierarchical relationships was very low, although the approval rate was good. Future work will focus on increasing their number.

Interestingly, we found that expert agreement for the first two uses cases was only fair. We think this is indicative of variability in clinical vocabulary based on experience. The medical student selected the smallest group of terms, while an attending physician selected the largest. An attending physician will have had more clinical experience and more chance to encounter wider clinical term usage.

Keeping the recommended list concise and similar in size across changes to the corpus size required adjusting the frequency threshold and corpus from which to recommend terms. As the number of clinical documents increased, the threshold frequency of occurrence for a recommended term could also increase, leading to a list with a 15% better approval rate. When the document corpus was highly specific the overlap between the clinical and biomedical corpora was too small causing us to widen the recommended term filter to include terms found only in the clinical text. This widening may have caused a lack of LSP and ASIUM results for the terms from clinical text only, leading to the low approval rates for the synonyms and hierarchical relationships.

One possible difficulty our recommended term list may have is due to the impact of partial matches both to UMLS and to the ontology terms. Term finding in Medicine is particularly complex because Medical vocabulary includes many multi-word terms [34]. Identifying the constituents of multi-word clinical concepts can be difficult. In some cases, a multi-word term containing an anatomic location is seen as a single term. For example, “perforation of the lower bowel” includes the UMLS concepts *(disorder) bowel perforation* and *(anatomical location) lower bowel*, *bowel* must be attached to both *perforation* and *lower* to find the appropriate mappings. Those words alone are not UMLS concepts. In contrast, “cranial fracture of the right parietal bone” includes the UMLS concepts *(disorder) fracture*, *(anatomical location) cranium* specifically *(anatomical location) right parietal bone*. Here *cranial* must be separated from *fracture* to identify the correct UMLS concept and two different anatomic locations must be reconciled. Because it is not possible for the machine to determine when words must be combined or split, terms that have overlapping words are considered matches. However, not all overlaps are equivalent. On the ACMS recommended term list *ca* is considered a match for ‘abdominal discomfort relieved by defe*ca*tion’, which is obviously wrong. A simple solution would appear to be to insist on spaces before or after the partially matched word. This solution would cause words partially matched due to plurals (e.g. *vital sign*, *vital signs*) to become misses. It would also interfere with finding terms that have been abbreviated in the clinical note. Clinical notes are prone to ad hoc abbreviations [51]. By including partial-word matches we hoped to catch “ca” used to refer to “cancer” or “roc” to “rocuronium”. We will address this problem further in future work. In pursuit of the solution, we will re-examine stemming. These types of terms are an example of why ontology developments systems are currently only semi-automated.

One of the reviewers pointed out that doing a head-to-head comparison with a system from the introduction would be a simple test of SEAM’s efficacy. However, we were unable to perform a test. Ontolearn is not available as software (documentation and ontology files can be downloaded). OntoLT is available but only compatible with the 2006 version of Protégé. Text2onto also dates back to 2007. A system that we could access was the OBO-edit term finder. This and other term finding systems are not performing the same task as SEAM. They seem to be aimed at extracting as many words as possible from a single document, not looking across documents for commonalities. The OBO-edit term finder, when given any one of the SEAM corpora, failed to return results. It did return a long list of results for a single document.

In their paper on challenges and new directions in biomedical ontologies, Hoehndorf, et al, describe four aspects of an ontology that should always be addressed [52]. The first aspect is the *degree of formality* of the language. SEAM supports formal ontology development. The larger SEAM system exports the ontologies created with it in OWL. The second aspect is the *complexity* of the ontology description. SEAM proposes descriptions from the source documents using simple lexical patterns. The descriptions found in this way are not complex. SEAM is limited in that a rich ontology should also include mierological (i.e. part of) relationships. There are plans to include a *part of* component for SEAM, but it is not yet functioning well enough to report. The third ontology aspect is the *interpretation* of what constitutes a *class*. This aspect of ontology refers to the decision to build using realist principals or not. Realist principals dictate that the concepts in the ontology represent entities in the real world. We leave *interpretation* decisions to human reviewers. We will include in the expanded SEAM system guidance for developers about creating realist entities and maintaining their integrity through the development process. The final aspect is the *orthogonality* of the context of the ontology. SEAM is designed to expand existing ontologies. The larger SEAM system is able to read in OWL files and expand the ontologies contained in them. However, ontology integration is not available in this lightweight iteration.

The realist aspect to the ontologies described here is the MUS top-level from [1]. One of the findings from the current work that will have an impact on the top-level comes from the qualitative terms in the recommended term sets. Terms like *was considered*, *revealed*, *consideration*, and *confirmed* were reminders that when reading a document one creates not only a representation of the world that it is describing, but also a representation of the document’s author. This representation of the author (e.g. are they decisive, are they reliable) allows the reader to interpret not just the world as the author saw it, but to extrapolate to something closer to the world as it actually is. We are currently pursuing a way to include concepts related to the representation of the author into an ontology for our larger goal of extracting knowledge from clinical documents.

### Future work

In future work, we will address a more advanced version of context vector synonymy calculations. We will weight the intersection of context vectors based on the number of times the matched word occurred for each term.

In a different setting, we will investigate the ontologies including their coverage of their target domain. We will also assess their utility in automated chart review to determine patients with MUS or ACMS and the expansion of the echo pick-list.

Expanding SEAM to domains outside of medicine by replacing journal articles with those from the new field of interest and clinical texts with other technical reports would be straightforward, but changing the dictionary look-up would be more difficult. We are not aware of a resource equivalent to the UMLS Metathesaurus for other specialized domains. WordNet could be used, but it seems likely to be too general to eliminate many irrelevant words. However, use case 3 shows that highly specialized texts may overcome the need for elimination through look up. Some developers have used textbook glossaries as specialized look-up sources [10]. Since our work is focused on medicine, we have not explored this issue in detail.

## Conclusion

SEAM can produce a concise list of recommended clinical terms, synonyms and hierarchical relationships regardless of medical domain. SEAM combines many term, synonym and relationship extraction techniques and uses combined clinical and biomedical corpora to target the relevant terms and relationships. We have demonstrated the generality of the system across three use cases: Two hand-created ontologies (one for acute changes in mental status (ACMS) and one for Medically Unexplained Syndromes (MUS)); and an ontology of echocardiogram summary statements.

### Availability and requirements

- **Project name:** SEAM
- **Project home page:**http://kdh-nlp.org/Seam-project/seam-home.html
- **Operating system(s):** Platform independent
- **Programming language:** Java (1.6 or higher)
- **Other requirements:** MySQL Metathesaurus
- **License:** GNU General Public License v3
- **Any restrictions to use by non-academics: See NOTICE in Software**

## Endnote

^a^Ontology entries are also referred to as entities. The distinction between *entities* and *concepts* reflects a current difference in the approach to ontology development that is outside the scope of this paper. Here we choose *concept* since we will not address the additional steps necessary to identify *entities*.

## Abbreviations

ACMS: Acute change in mental status
ECHO: Echocardiogram
I2b2: Informatics for integration biology and the bedside
IE: Information extraction
LSP: Lexico-syntactic pattern
MUS: Medically unexplained syndrome
NER: Named entity recognition
NLP: Natural language processing
NP: Noun phrase
OWL: Web ontology language
PDF: Portable document format
TF-IDF: Term frequency by inverse document frequency
UMLS: Unified medical language system
UMLS CUI: UMLS metathesaurus concept unique identifier
UMLS TUI: UMLS metathesaurus (Semantic) type unique identifier
XML: Extensible markup language
